# 561. Phase 3 Trial of Fostamatinib for the Treatment of COVID-19: Repurposing an Immunomodulatory Drug Previously Approved for Immune Thrombocytopenia

**DOI:** 10.1093/ofid/ofab466.759

**Published:** 2021-12-04

**Authors:** Ziad Mallat, Suzana Margareth Lobo, Anuj Malik, Sandra Tong

**Affiliations:** 1 University of Cambridge, Cambridge, UK; 2 Hospital De Base -FAMERP, São José do Rio Preto, Sao Paulo, Brazil; 3 Ascension St. John Medical Center, Tulsa, Oklahoma; 4 Rigel Pharmaceuticals, Inc., South San Francisco, CA

## Abstract

**Background:**

Key pathologies in severe COVID-19 include immune cell activation, inflammatory cytokine release, and neutrophil extracellular trap release (NETosis), which are mediated by spleen tyrosine kinase (SYK) (Figure 1). Fostamatinib, an oral SYK inhibitor approved for chronic immune thrombocytopenia, has shown activity in vitro using plasma from patients with severe COVID-19, by abrogating the hyperimmune response triggered by anti-spike IgG; ^1^ inhibiting hyperactivation in platelets; ^2^ and blocking NETosis in neutrophils.^3^ R406, active metabolite of fostamatinib, protected against LPS-induced acute lung injury and thrombosis in mice.^4,5^ In clinical studies, fostamatinib reduced IL-6 in patients with rheumatoid arthritis.^6^ Therefore, a phase 2 study (NCT04579393) evaluated fostamatinib vs. placebo plus standard of care (SOC) in 59 hospitalized COVID-19 patients (manuscript pending). We initiated a phase 3 clinical study (NCT04629703) of fostamatinib for the treatment of COVID-19.

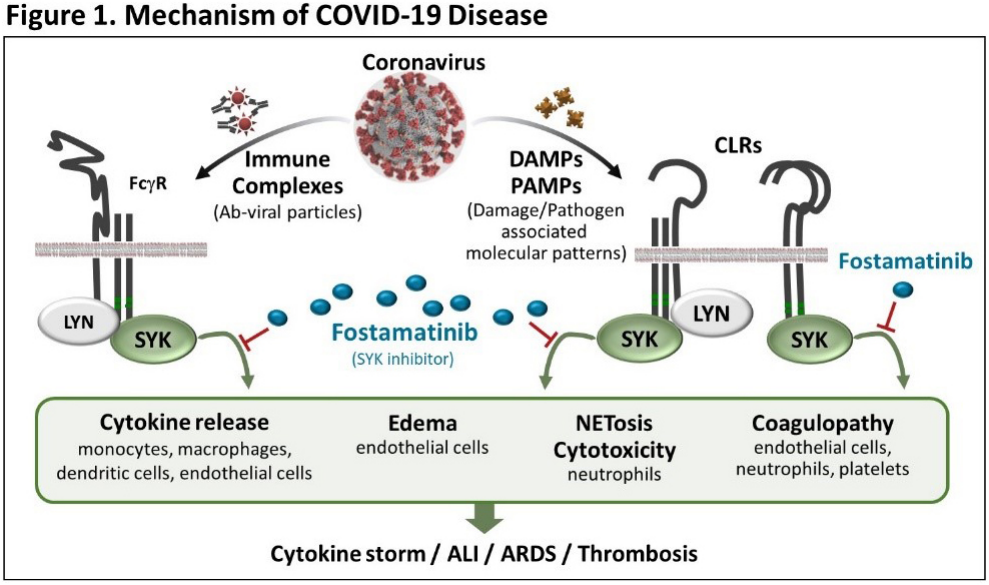

**Methods:**

A double-blind, randomized, placebo-controlled, adaptive design, multi-center, Phase 3 study (NCT04629703) is underway to evaluate the safety and efficacy of fostamatinib in 308 adult patients with COVID-19 (Figure 2). Hospitalized patients without respiratory failure (with or without supplemental oxygen) were included. Patients with ARDS or using extracorporeal membrane oxygenation (ECMO) were excluded. Patients will receive fostamatinib 150 mg BID or placebo for 14 days; both arms receive SOC. The primary outcome will be progression to severe/critical disease (worsening in clinical status score on the 8-point ordinal scale) within 29 days of the first dose of study drug. Fostamatinib is investigational for COVID-19.

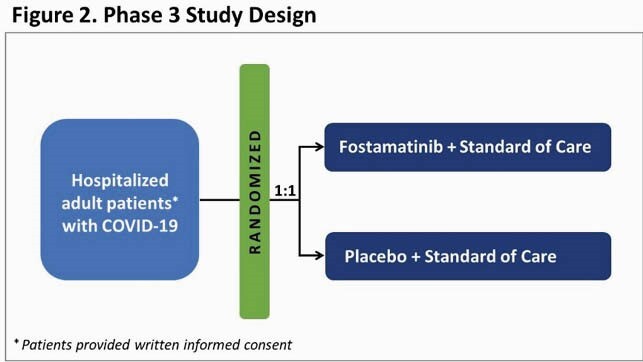

**Results:**

Blinded update of trial in progress as of 28 April 2021. 12 patients have been randomized in North and South America. The clinical status score at Baseline was 5 (Hospitalized, requiring supplemental oxygen) in all 12 patients. Five patients had 8 adverse events (AE) (Fig 3). One AE (PE) was serious and is resolving. No deaths have been reported. At least two patients have been discharged (Day 5, Day 13) with continued dosing at home.

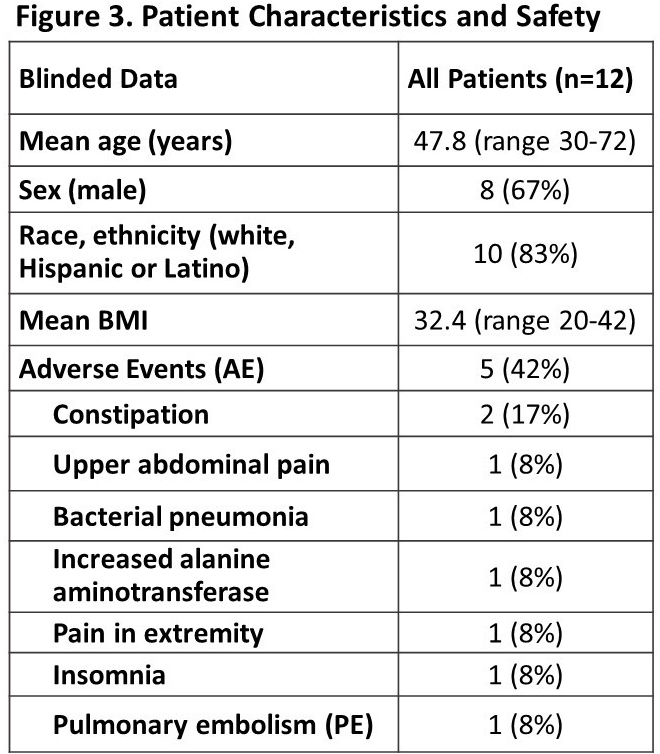

**Conclusion:**

Fostamatinib has the potential to provide a treatment option for the hyperimmune complications of COVID-19.

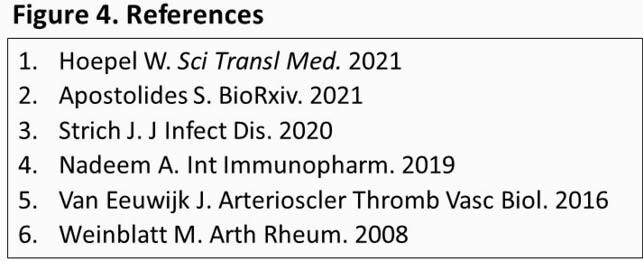

**Disclosures:**

**Ziad Mallat, MD, PhD**, Rigel Pharmaceuticals, Inc. (Consultant) **Sandra Tong, MD**, Rigel Pharmaceuticals, Inc. (Employee, Shareholder)

